# Subthalamic Nucleus Subregion Stimulation Modulates Inhibitory Control

**DOI:** 10.1093/texcom/tgaa083

**Published:** 2020-11-04

**Authors:** Nelleke C van Wouwe, Joseph S Neimat, Wery P M van den Wildenberg, Shelby B Hughes, Alexander M Lopez, Fenna T Phibbs, Jeffrey D Schall, William J Rodriguez, Elise B Bradley, Benoit M Dawant, Scott A Wylie

**Affiliations:** 1 Department of Neurological Surgery, University of Louisville, Louisville, KY 40202 USA; 2 Department of Neurology, Vanderbilt University Medical Center, Nashville, TN 37232, USA; 3 Department of Psychology, University of Amsterdam, Amsterdam 1018 WS, The Netherlands; 4 Amsterdam Brain and Cognition (ABC), University of Amsterdam, Amsterdam 1001 NK, The Netherlands; 5 Department of Psychology, Vanderbilt University, Nashville, TN 37240, USA; 6 Department of Electrical Engineering and Computer Science, Vanderbilt University, Nashville, TN 37235, USA

**Keywords:** deep brain stimulation, inhibitory action control, Parkinson’s disease, subthalamic nucleus

## Abstract

Patients with Parkinson’s disease (PD) often experience reductions in the proficiency to inhibit actions. The motor symptoms of PD can be effectively treated with deep brain stimulation (DBS) of the subthalamic nucleus (STN), a key structure in the frontal–striatal network that may be directly involved in regulating inhibitory control. However, the precise role of the STN in stopping control is unclear. The STN consists of functional subterritories linked to dissociable cortical networks, although the boundaries of the subregions are still under debate. We investigated whether stimulating the dorsal and ventral subregions of the STN would show dissociable effects on ability to stop. We studied 12 PD patients with STN DBS. Patients with two adjacent contacts positioned within the bounds of the dorsal and ventral STN completed two testing sessions (OFF medication) with low amplitude stimulation (0.4 mA) at either the dorsal or ventral contacts bilaterally, while performing the stop task. Ventral, but not dorsal, DBS improved stopping latencies. Go reactions were similar between dorsal and ventral DBS STN. Stimulation in the ventral, but not dorsal, subregion of the STN improved stopping speed, confirming the involvement of the STN in stopping control and supporting the STN functional subregions.

## Introduction

Abruptly stopping our actions when they are no longer adaptive is a critical component of executive cognitive control to navigate dynamic environments flexibly and safely (Ridderinkhof et al. 2011; Mirabella 2014). Reductions in the proficiency to inhibit actions are reported in several neurological and neuropsychiatric disorders with altered function of the neural circuitry linked to inhibitory action control: the frontal–basal ganglia network (Alderson et al. 2007; Wylie et al. 2016; Manza et al. 2017). The subthalamic nucleus (STN) is a key structure in the broader frontal–striatal network that may be directly involved in regulating inhibitory control (Aron and Poldrack 2006; Aron et al. 2007; van den Wildenberg et al. 2010; Zandbelt and Vink 2010; Forstmann et al. 2012; Jahanshahi et al. 2015; Aron et al. 2016), including the striatum, the globus pallidus, the cerebellum, primary motor cortex, and premotor cortex (Coxon et al. 2006; Li et al. 2008; Zandbelt and Vink 2010; Mirabella et al. 2011; Mattia et al. 2012; Brunamonti et al. 2014; Mallet et al. 2016). Current models propose that the STN suppresses the basal ganglia output to the cortex, which functionally stops response-generating signals from activating motor actions (Nambu et al. 2002; Bogacz and Gurney 2007; Wiecki and Frank 2013).

The organization of cortical afferents to STN creates functional specialization within STN subregions, which could be specifically relevant for the implementation of inhibitory control. Direct evidence supporting the role of a specific STN subregion to inhibitory stopping control is however limited. The presupplementary motor area (preSMA) and the inferior frontal cortex (IFC) are among the most commonly proposed cortical areas linked to stopping control, and both regions send converging projections to a relatively more ventral subregion of the STN (Haynes and Haber 2013; Aron et al. 2016) (but see Schall and Godlove 2012; Erika-Florence et al. 2014; Thunberg et al. 2020 for counterperspectives on cortical areas involved in stopping control).

Given the apparent dissociation of cortical inputs to STN subregions, we tested whether a relatively more ventral STN subregion is critical to the human stopping control by applying focused stimulation to this subregion and separately to a relatively more dorsal STN subregion, while the participants completed the stop-signal task, which measures the inhibitory stopping control.

## Modulating Inhibitory Control with STN DBS

Deep brain stimulation (DBS) to the STN is an important treatment option for the cardinal motor symptoms in advanced Parkinson’s disease (PD). Each DBS electrode has multiple contact points that traverse the STN and can be leveraged to stimulate different subterritories of the STN to test functional modulation. Clinically, stimulation generally targets a contact point in the most dorsal “motor” subregion of the STN, which is innervated by cortical projections from the primary motor cortex (M1) and supplementary motor area (SMA) (Haynes and Haber 2013; Plantinga et al. 2018). Stimulation in this subregion is thought to ameliorate pathological network oscillations that lead to PD motor symptoms (e.g., bradykinesia, tremor, and rigidity) (Herrington et al. 2016). However, clinical stimulation settings produce large tissue activation fields that impact a substantial part of the STN and surrounding structures, which likely explains the presence of cognitive and emotional effects (beneficial or disruptive effects like hypomania and depression) (Mallet et al. 2007; Okun et al. 2009; Accolla and Pollo 2019) when stimulating in the STN “cognitive” and “limbic” regions, which are located relatively ventral from the dorsal motor region.

Moving ventrally from the dorsal motor subregion toward the center of the STN nucleus reveals a different pattern of cortical afferents characterized by converging projections from preSMA, IFC, and dorsolateral prefrontal cortex (DLPFC) (Haynes and Haber 2013). Imaging studies suggest that the preSMA, IFC, and STN form a network involved in inhibitory stopping control (Aron et al. 2004; Forstmann et al. 2012; Zandbelt et al. 2013; Aron et al. 2014). Supporting this hypothesis are neurophysiological studies showing increased power in the beta frequency (13–30 Hz) in the STN coincident with stopping (Kuhn et al. 2004; Ray et al. 2012; Alegre et al. 2013; Bastin et al. 2014) and increased spiking activity in STN with successfully stopped responses (Isoda and Hikosaka 2008; Bastin et al. 2014). In a recent primate study, single-unit activity in a more ventral STN subregion was linked directly with stopping (Pasquereau and Turner 2017). Like the modulation of beta power with stopping in the STN, cortical electroencephalography studies reported increased beta power in right IFC and preSMA coincident with stopping (Swann et al. 2009; Swann et al. 2012; Wessel et al. 2013), or increased coherence between IFG and STN with faster stopping (Chen et al. 2020). These studies lend to the hypothesis that IFC/preSMA and their projections to a ventral STN subregion are implicated in stopping control. However, there is limited evidence linking stimulation along the STN dorsal–ventral axis to dissociable effects on inhibitory control.

Studies of PD patients treated with STN DBS provide an opportunity to investigate the effects of direct stimulation to STN on stopping control. Initial studies testing the effect of clinical stimulation provided support that stimulating the STN with broad stimulation fields improved stopping latency (i.e., shorter stop-signal reaction times [SSRTs]) in PD patients (van den Wildenberg et al. 2006; Swann et al. 2011; Mirabella et al. 2012), but see Obeso et al. (2013) for slower SSRTs with DBS on a conditional stop task and with unilateral DBS in the left STN (Ray et al. 2009) and the absence of an effect with unilateral DBS (Mancini et al. 2018). Two studies (Hershey et al. 2010; Greenhouse et al. 2011) examined stimulation along the dorsal–ventral axis through different contact points of the DBS electrode on inhibitory control; one reported no dissociable effects on stopping (Greenhouse et al. 2011), and the other reported increased commission errors on a Go–NoGo task with unilateral stimulation targeting a relatively more ventral STN subregion (Hershey et al. 2010). However, both studies used broad stimulation fields (with clinical DBS) and contact points at the extreme ends of the DBS electrode with several outside of the STN. Our previous work with focused DBS in the STN subregions on a Simon conflict task showed that DBS in the dorsal STN, but not in the ventral STN subregion, improved the selective inhibition of conflicting action impulses (van Wouwe et al. 2017). This finding (focal DBS in the dorsal STN improves selective inhibition) and the neurophysiological and imaging studies linking the ventral STN circuitry to stopping (Aron et al. 2016; Pasquereau and Turner 2017) suggest that there might be a functional dissociation for inhibitory control across the STN. However, there has not been a precise test of the hypothesis that focused stimulation of specific STN subregions produces dissociable effects on stopping control.

In the current study, we investigated the “causal” effect of focused STN stimulation on inhibitory control across dissociable STN subregions in a group of PD patients treated with STN DBS. We turned OFF clinical stimulation settings and applied focused (subtherapeutic) stimulation parameters to restrict the projected field of tissue activation to a circumscribed dorsal or ventral STN subregion. Participants performed the stop-signal task once with bilateral dorsal and once with bilateral ventral STN stimulation. The stop-signal task yields an estimate of an individual’s stopping latency (SSRT), and longer SSRTs (i.e., slower stopping) reflect poorer proficiency at inhibiting actions (Bissett and Logan 2011).

Given existing evidence that key cortical regions linked to stopping project to a relatively ventral STN subregion and STN stimulation at clinical settings improves stopping latency, we predicted that focused stimulation delivered to this specific STN subregion would improve stopping (i.e., faster SSRTs) compared with stimulation in a dorsal STN subregion.

## Materials and Methods

### Participants

PD participants (*n* = 24), after a minimum of 6 months of treatment with bilateral STN DBS, were recruited from the Vanderbilt University Medical Center Neurology and Functional Neurosurgery clinics. To limit the duration of the testing time OFF medication and OFF clinical stimulation for participants, one group of DBS patients (*n* = 12) participated in the stimulation procedure (dorsal and ventral DBS), whereas another control group of DBS patients (*n* = 12) participated in the OFF stimulation condition only. All 24 patients were withdrawn from their dopaminergic medication during participation.

Participants were excluded from recruitment if they had history of: (1) comorbid neurological condition(s) other than PD (e.g., essential tremor), (2) bipolar affective disorder or schizophrenia, (3) severe, treatment-resistant mood disorder, or (4) other medical condition directly impacting cognitive functions (e.g., cardiac condition, and pulmonary disease). Participants with a history of depression or anxiety were allowed to participate if their symptoms were treated, stable, and of mild or low moderate severity at study entry (i.e., similar to requirements for surgical candidacy), determined by consensus conference reviews, neuropsychological interviews, and questionnaires (Center for Epidemiologic Studies Depression Scale [CESD], Radloff 1977). Participants were allowed mild cognitive difficulties, that is, a Mini Mental State Examination (MMSE) score of 25 or higher Folstein et al. 1975), but they were excluded if their neuropsychological testing indicated early stage dementia. All participants reported corrected-to-normal vision.

Enrolled PD participants underwent neurosurgical DBS using standard stereotactic techniques coupled with microelectrode recordings and intraoperative motor testing to optimize contact placement, see Konrad et al. (2011). Participants were implanted with an Activa PC Medtronic neurostimulator (Medtronic Inc.), and they showed post-DBS improvements in their clinical motor symptoms for at least 6 months as determined by the medical record review and neurological ratings of motor symptoms (Unified Parkinson’s Disease Rating Scale Motor [UPDRS]). See Table 1 for mean demographics and clinical information of the participants.

**Table 1:** Demographic data (means and SD) for the PD DBS patients from the stimulation group (DBS ON; group that received both dorsal and ventral DBS) and the DBS OFF group (control patients OFF stimulation)

	Demographics		*F*-value	*P* value
	DBS ON (mean dorsal/ventral DBS group)	DBS OFF (mean OFF DBS group)	DBS ON versus DBS OFF	
Sample size (*N*)	12	12		
Age (years)	58.75 (8.09)	62.33 (10.26)	1.33	0.26
Sex (M:F)	7:5	7:5		
Education (years)	15.13 (3.64)	15 (3.16)	0.02	0.88
MMSE	28.58 (1.56)	28.25 (2.05)	0.36	0.56
CESD	14.33 (9.13)	13.25 (8.69)	0.20	0.66
BIS II	60.92 (11.63)	59.92 (8.76)	0.23	0.64
LEDD	450.83 (433.50)	612.92 (350.17)	1.01	0.33
Disease duration (years) (years) +	13.83 (7.70)	10.92 (6.44)	1.10	0.31
UPDRS dorsal	29.50 (8.34)		2.3^a^	0.14
UPDRS ventral	29.25 (10.64)		1.64^b^	0.21
UPDRS OFF		24.58 (9.27)		

Note: SD, standard deviation; BIS II, Behavioral ImpulsivityII.

^a^Dorsal DBS versus OFFDBS.

^b^Ventral DBS versus OFFDBS.

Participants provided informed consent prior to enrollment and the research was performed in full compliance with the standards of ethical conduct in human investigation as regulated by the Vanderbilt University. Enrolled participants were taking dopaminergic medication, see Table 1 for converted levodopa equivalent daily dose (LEDD, Tomlinson et al. 2010); but all participants (*n* = 24) completed the study during a single visit in an OFF dopamine medication state following a 24-h withdrawal from levodopa and 48-h withdrawal from dopamine agonist.

### DBS Contact Registration and Selection

Participants considered for the study underwent a preoperative brain magnetic resonance imaging (MRI) (*T*1-weighted and *T*2-weighted) and a 1-month postoperative brain computerized tomography (CT) as a part of the standard clinical care. The MRI was acquired with a 3T Philips (Philips Achieva) using phased-array SENSE 8-channel reception and body coil transmission. *T*1-weighted images (typical time repetition [TR]/time echo [TE] = 7.9/3.6 ms) were captured with 1.0 mm^3^ isotropic spatial resolution, and *T*2-weighted images (typical TR/TE =3000/80 ms) were captured with a 47 × 47 mm^2^ in-plane resolution and 2 mm slice thickness. CT images were acquired at kVp = 120 V, with 350 mAs exposure, capturing 512 × 412 pixels. In-plane resolution and slice thickness were set at approximately 0.5 and 0.75 mm, respectively.

**Figure 1:**
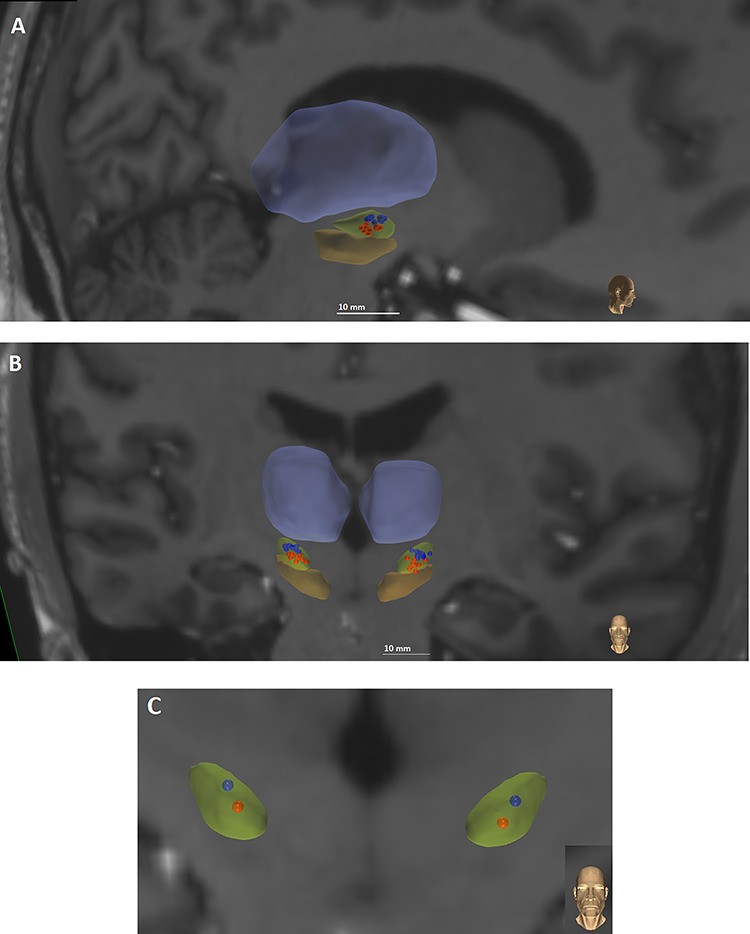
Individual electrode positions in STN (green) used for dorsal (blue) and ventral (red) stimulation in sagittal (*A*) and coronal (*B*) planes and (*C*) average electrode position in coronal plane. Substantia nigra and thalamus are displayed for reference in orange and violet.

DBS contact localization for each participant and projection onto a brain atlas, that is, a reference brain volume, was done using methods similar to those described by van Wouwe et al. (2017). The CranialVault Explorer (CRAVE) Software (D'Haese et al. 2012) was used to automatically localize the implants and individual contacts in the CT images. Automatic localization was subsequently verified visually and contact position was adjusted if necessary. Preoperative MRIs and postoperative CTs were registered using fully automatic intensity-based rigid registration techniques integrated into CRAVE. These steps allowed for the visualization of individual contacts on the anatomical MRI images of the patient. The preoperative MRI was registered to the brain atlas in which deep brain anatomic structures are segmented using high field (7 Tesla) images (Liu et al. 2020). Registration was performed with a fully automatic intensity-based nonlinear image registration technique that was also integrated into Crave (Rohde et al. 2003). The accuracy of the registrations was assessed visually for each volume. This process allowed the projection of individual contacts onto the segmented atlas STN. The ventral and dorsal region of the STN were defined using an oblique plane (perpendicular to the lead trajectory) to divide dorsolateral and ventromedial subregions, along the lines of Hayes and Haber (Haynes and Haber 2013). We used the same subdivision in our previous work (van Wouwe et al. 2017). Participants were recruited into the study if bilateral leads had at least one contact centered in the dorsal and one contact centered in the ventral subregions of the STN. Figure 1*A*,*B* displays the individual participant electrode contacts distributed in the dorsal and ventral regions of the STN, and Figure 1*C* shows the average contact location across participants.

### Design and Procedures

One group of participants (*n* = 12) completed two sessions of the stop-signal task, once with bilateral STN DBS at dorsal and once at ventral contacts. The other group of participants (*n* = 12) performed the stop-signal task without stimulation (one session).

Participants in the stimulation procedure were blind to the order of stimulation targets across sessions, which was counterbalanced across the participants. After a DBS setting change (i.e., before the first testing session and between sessions), we imposed a 30-min waiting period before starting cognitive testing. Our previous work suggests that this time period is sufficient to find effects on inhibitory control (van Wouwe et al. 2017), and this time period also accounts for most of the changes in the motor symptoms (Lopiano et al. 2003; Temperli et al. 2003). See Tables 2 and 3 for the patients’ clinical settings and the electrodes used with experimental dorsal and ventral stimulations.

**Table 2:** Clinical stimulation settings (mean and SD) for both patient groups, that is, the DBS ON (stimulation group that received both dorsal and ventral DBS) and the DBS OFF group (control patients OFF stimulation)

	Clinical DBS settings	
	DBS ON (mean dorsal/ventral DBS group)	DBS OFF (mean OFF DBS group)
Sample size (*N*)	12	12
Left		
Voltage (V)	2.51 (0.78)	2.48 (0.99)
Frequency (Hz)	126.67 (8.88)	129.17 (2.89)
Pulse width (ms)	59.08 (15.29)	65.83 (11.65)
Right		
Voltage (V)	2.23 (0.83)	2.53 (1.19)
Frequency (Hz)	126.67 (8.88)	129.17 (2.87)
Pulse width (ms)	61.58 (17.71)	70.00 (13.48)

**Table 3:** Electrodes used for stimulation at dorsal and ventral contacts

Subject ID	Dorsal contact		Ventral contact	
	Left	Right	Left	Right
**1**	3	3	2	2
**2**	3	3	2	2
**3**	2	2	1	1
**4**	2	3	0	2
**5**	2	3	1	2
**6**	1	1	0	0
**7**	3	2	2	1
**8**	2	2	1	1
**9**	2	2	1	1
**10**	3	3	2	2
**11**	2	2	0	0
**12**	3	2	1	0

Note: Experimental bilateral stimulation settings were set at 0.4 mA, 130 Hz frequency, and 60 ms pulse width.

For Medtronic 3389 leads, 0 indicates the most ventral lead, and 3 is the most dorsal lead of the four-contact array. The 3389 leads have an electrode contact size of 1.5 mm with 0.5 mm spacing between contacts. For subjects 4 (left side), 11, and 12, ventral and dorsal stimulations were not at adjacent contacts, whereas the other participants received stimulation at a ventral contact immediately below a dorsal contact. However, the direction of results on SSRT is similar for these three subjects, showing faster SSRTs with ventral compared with dorsal DBS (mean SSRT_dorsal_ = 326 ms, mean SSRT_ventral_ = 237ms).

The stimulation parameters were altered from the participants’ clinical settings to isolate the targeted subregions of the STN. Because clinical DBS settings often vary across individuals and involve parameters that produce large stimulation fields (overlapping across adjacent contacts), we instead restricted stimulation to a constant current of 0.4 mA while holding the stimulation frequency at 130 Hz and pulse width at 60 μs (van Wouwe et al. 2017). These settings provided (to the extent possible) a uniform current density across the targeted STN subregions and across participants, while also restricting the estimated field of tissue activation. Based on Butson and McIntyre (2008), a stimulation amplitude of approximately 0.4 mA (with an average clinical impedance of 1 kΩ) would result in a radius of the volume of activated tissue (VTA) of about 1.3 mm. Similar fields from adjacent electrodes would have a minimal volumetric overlap, suggesting discernable effects.

### Stop-Signal Task

Participants completed an arrow-based stop-signal task delivered on a 15-inch laptop, placed approximately at 90 cm in front of the participant. Participants viewed left or right pointing, gray-colored arrows (Go stimuli) presented one at a time against a white-colored background, and they responded to each arrow with a manual left- or right-thumb button press using handheld grips. A fixation point (square) remained on the screen across the task and was visible between the trials. Participants were instructed to focus on the fixation point, and when the arrow appeared, to press the button as quickly and as accurately as possible with the hand similar to the direction indicated by the arrow (left-pointing arrow = left button; right-pointing arrow = right button). Arrows remained on the screen until a response was issued or 1500 ms elapsed. After a response was made, a variable interval between 1750 and 2250 transpired before the onset of the next arrow.

On 25% of the trials, the arrow would unpredictably change to purple after a brief delay. This color change served as a stop-signal that instructed the participant to attempt to stop their button press response. For these stop-signal trials, the delay between the initial onset of the gray arrow and the color change adjusted dynamically using a 50 ms staircase-tracking procedure based on the participant’s success or failure at stopping on the previous stop-signal trial (Levitt 1971), see Hughes et al. (2019) for more details. This dynamic tracking procedure converges to 50% stopping success, which is a requisite for computing a reliable estimate of a participant’s SSRT (Band et al. 2003). Go stimuli and stop-signal stimuli occurred randomly and with equiprobability for left- and right-pointing arrows. With each cognitive testing session, participants in the stimulation group practiced 48 trials before completing 2 experimental blocks of 104 trials (208 total experimental trials: 156 Go, 52 stop). Participants in the OFF stimulation group performed the Stop task with a slightly different number of trials, that is, they practiced 60 trials and performed 2 experimental blocks of 120 trials (240 total: 180 Go, 60 stop).

### Analyses

First, Go RT and square-root-transformed Go accuracy rates were compared between dorsal and ventral stimulations. We performed one-way ANOVAs to compare Go RT and accuracy rate differences between the OFF and each stimulation condition (comparing the DBS OFF group separately with the ventral and dorsal DBS) and paired sampled *t*-tests to compare Go RT and accuracy rate differences between the dorsal and ventral STN stimulations (within the group of participants receiving dorsal and ventralDBS).

Second, one-way ANOVAs were used to compare stopping latency (SSRT) differences between the OFF and each stimulation condition (we again used separate comparisons to contrast OFF DBS with the ventral and dorsal DBS) and paired sampled *t*-tests to compare stopping latency differences between ventral or dorsal STN stimulation. SSRT was calculated based on the horse race model and integration method (Logan 1994; Verbruggen and Logan 2009).

We verified critical assumptions of the horse race model requiring that average RTs of responses on stop-signal trials that failed inhibition were shorter than average Go RTs (Logan 1994; Band et al. 2003) and that Go RTs were uncorrelated with SSRTs. Statistical computations were performed in SPSS (Version 26,IBM).

To provide additional quantification of the strength of our findings (Wagenmakers 2007), the main hypotheses were also examined by calculating a Bayes factor (Rouder et al. 2012; Wetzels et al. 2012; Jarosz and Wiley 2014). The Bayes factor (BF_10_) provides the odds ratio for the alternative versus the null hypotheses, given a particular data set. A value of 1 means that null and alternative are equally likely, larger values suggest that the data are in favor of the alternative hypothesis, and smaller values (<1) indicate that the data are in favor the null hypothesis. More specifically, BF10 values larger than 3 are considered as moderate support, and BF10 values greater than 10 are considered as strong support, for the alternative hypothesis. Conversely, BF10 values smaller than 0.3 or smaller than 0.1 provide moderate-to-strong support for the null hypothesis (Jeffreys 1961). We used JASP 0.11.1.0 (Love et al. 2015) to calculate the Bayes factor.

## Results

### Go RTs

**Figure 2:**
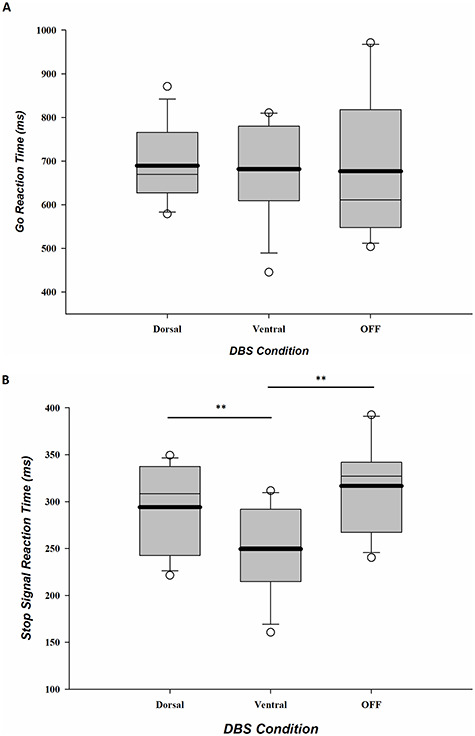
Medians, means (bold line), and 95% confidence intervals for Go RT (*A*) and SSRTs (*B*) separate for dorsal and ventral STN stimulation conditions and for the OFF stimulation group. Significant differences are indicated with an asterisk, ^*^P < 0.05, ^**^P < 0.01.

Table 4 shows the mean performance measures and statistics on the stop-signal task for each group and stimulation condition. Figure 2*A* shows the mean Go RTs for each group and stimulation condition.

**Table 4:** Mean and SD of stop-signal performance with dorsal and ventral STN stimulation

	Dorsal DBS	Ventral DBS	OFF DBS	*t*-Value	*F*-Value	
				Dorsal–ventral	Dorsal-DBS OFF	Ventral-DBS OFF
Go RT (ms)	690 (88)	682 (108)	678 (165)	0.41	0.06	0.008
Go errors (%)	3.7 (3.4)	2.5 (2.8)	2.3 (3.3)	1.53	1.14	0.15
Go omission errors (%)	1.7 (2.6)	2.5 (6.5)	2.4 (5.1)	0.41	0.18	0.001
Stop-signal delay (ms)	342 (73)	395 (136)	334 (148)	1.82	0.02	1.09
Signal respond rate (%)	43.4 (3.6)	45.5 (7.2)	48.5 (6.5)	0.84	5.62*	1.12
Signal respond RT (ms)	594 (65)	608 (88)	597 (165)	0.66	0.004	0.04
Stop-signal RT (ms)	294 (48)	250 (47)	317(49)	3.64**	1.31	11.74**

Note: Statistical comparisons include *t*-values from paired sampled *t*-tests (within-subject comparison dorsal–ventral DBS) and *F*-values from the one-way ANOVA’s comparing dorsal DBS versus OFF and ventral DBS versus OFFDBS.

^*^ *P* < 0.05

^**^ *P* < 0.01

### Stimulation Conditions Versus OFF DBS

As shown in Figure 2*A*, mean Go RTs and error rates under dorsal stimulation and ventral stimulation were not significantly different from performance in the OFF stimulation group (OFF-ventral: RT and ACC: *F*s < 1, *P*s > 0.7, ηs^2^ < 0.01, BFs10 < 0.5; OFF-dorsal: RT and ACC: *F*s < 1.2, *P*s > 0.2, ηs^2^ < 0.05, BFs10 < 0.6). The small Bayes factors (<1) favor the null hypothesis that there is no differential effect of stimulation (either dorsal or ventral) on Go RTs and Go accuracy rates in comparison to the performance by the group without stimulation.

### Dorsal Versus Ventral DBS

Mean Go RTs and error rates under dorsal stimulation were not significantly different compared with ventral stimulation (RT and ACC, *t*s < 1.6, *P*s > 0.15, Cohen’s *d*s < 0.4, BFs10 < 0.8). The small Bayes factors (<1) additionally confirm that the data favor the null hypothesis that stimulating dorsal and ventral STN subregions produced no differential effect on Go RTs and Go accuracy rates.

### Stop-Signal Dynamics

The tracking algorithm produced stopping success that approached 50% under both dorsal, ventral, and OFF stimulation conditions, which is within the recommended 25–75% range for estimating SSRTs (note that the dorsal stimulation condition had significantly lower stopping success rates compared to the OFF condition, *F*(1, 22) = 5.62, *P* = 0.03, and η2 = 0.20. Stopping success rates between the other conditions were not different though, *P*s > 0.3, and all subjects had stop success rates within the recommended range for SSRT calculation, that is, between 25% and 75% [OFF: min 38%, max 60 %; ventral: min 38%, max 65%; and dorsal: min 38%, max 50%]) (Verbruggen et al. 2019) and verifies the first requirement of the horse race model (Logan 1994). Consistent with a second requirement, reactions on stop-signal trials (i.e., when participants failed to inhibit their response) were associated with faster responses than reactions to pure Go stimulus trials.

We verified this assumption separately for each stimulation condition; RTs for responses escaping inhibition on stop trials were significantly faster than RTs on pure Go trials under dorsal (difference of 96 ms, *t*(11) = 5.96, *P* < 0.001, Cohen’s *d* = 1.72, BF_10_ = 303), ventral stimulation (difference of 74 ms, *t*(11) = 6.45, *P* < 0.001, Cohen’s *d* = 1.86, BF_10_ = 552), and OFF stimulation (difference of 80 ms, *t*(11)= 8.83, *P* < 0.001, Cohen’s *d* = 2.55, BF_10_ = 7250) (all 24 individual subjects conformed with the requirements of the horse race model).

Finally, the data confirmed a third requirement; go and stop processes were independent under both DBS conditions, that is, there was no correlation between Go RTs and SSRTs (Spearman’s rho_dorsal_ = 0.29, *P* = 0.37, BF10 dorsal = 0.38; Spearman’s rho_ventral_ = 0.31 , *P* = 0.33, BF10 = 1.1; Spearman’s rho_OFF_ = 0.11 , *P* = 0.75, BF10 = 0.57). With the horse race model key assumptions satisfied, we next analyzed the stopping latency (SSRT) differences between stimulation conditions.

### Stimulation Conditions Versus OFF DBS

Figure 2*B* shows the mean SSRTs for each group and stimulation condition and indicates that both dorsal and ventral STN stimulation have shorter stopping latencies relative to the OFF stimulation condition. However, only ventral stimulation significantly improved stopping latencies with 67 ms (*F*(1, 22)= 11.74, *P* < 0.01, η^2^ = 0.35, BF_10_ = 15.16). The Bayes factor (>10) corroborated this and provided strong evidence in favor of an effect of ventral stimulation on SSRT, that is, the evidence favoring the alternative hypothesis of a real difference was 15.16 times stronger than for the null hypothesis. Stopping latencies with dorsal stimulation were not significantly different compared with stopping latencies OFF stimulation (*F*(1, 22) = 1.31, *P* = 0.27, η^2^ = 0.06, BF_10_ = 0.60).

### Dorsal Versus Ventral DBS

As shown in Figure 2*B*, stimulating distinct subregions of the STN produced a dissociable effect on mean stopping latencies (*t*(11) = 3.64, *P* < 0.005, Cohen’s *d* = 1.04, BF10 = 12.8). Specifically, stimulation delivered through the ventral STN contacts was associated with significantly faster stopping latencies compared with stimulation delivered through dorsal STN contacts, a difference of 44 ms (note that there was no difference between left- and the right-hand SSRTs [*F*(1, 11) = 0.37, *P* = 0.56], nor an interaction of DBS STN subregion with response hand [*F*(1, 11) = 0.03, *P* = 0.88]). The Bayes factor confirmed strong evidence in favor of a dissociable effect of stimulation on SSRT (i.e., evidence favoring the alternative hypothesis of a real difference was 12.8 times stronger than for the null hypothesis).

## Discussion

The STN is a key node in the cortico–striatal network putatively involved in inhibitory control. Recent studies have suggested that the IFC/preSMA projections to a relatively more ventral STN subregion may be implicated directly in stopping control (Aron et al. 2016; Pasquereau and Turner 2017). We predicted that focused stimulation delivered to the ventral STN subregion would improve stopping control compared with stimulating the most dorsal STN subregion.

Performance of PD patients on the stop-signal task satisfied the requirements of the horse race model, producing reliable and interpretable estimates of stopping latencies and Go RTs. Stimulating ventral and dorsal subregions of the STN produced dissociable effects on stopping. Bilateral stimulation of the ventral STN produced faster stopping latencies compared with stimulating the dorsal STN subregion and compared with the condition without stimulation Go RTs and errors, and on the other hand, remained similar between the subregion stimulation and without stimulation. This provides new, causal evidence that the modulatory effect of stimulation on stopping control depends on the STN subregion and confirms the importance of the ventral STN in modulating stopping control.

### STN Subregion Versus Clinical DBS Effects on Stopping

Previous studies using the stop task in PD showed that compared with DBS OFF conditions, applying DBS at clinical settings improves the proficiency of inhibiting actions (van den Wildenberg et al. 2006; Swann et al. 2011; Mirabella et al. 2012). The current study included two within-subject stimulation conditions (dorsal DBS vs. ventral DBS) and an additional separate group of participants, which performed the task OFF DBS (control condition) to keep the experimental time manageable for patients. Ventral STN stimulation produced a beneficial effect relative to dorsal STN stimulation and to the condition without stimulation. Stopping speed with ventral stimulation (SSRT = 250 ms) was comparable to values reported previously in an ON (clinical) DBS state, which ranged from 230 to 283 ms (van den Wildenberg et al. 2006; Swann 2011; Mirabella et al. 2012). By contrast, OFF DBS stopping latencies in those studies ranged from 285 to 311 ms, aligning with stopping in the dorsal stimulation and OFF stimulation condition in our study (SSRT_dorsal_ = 294 ms, SSRT_OFF_ =317 ms). Since the focal dorsal stimulation in the current study is comparable to OFF stimulation, this would suggest that if the VTA induced by clinical DBS moves too far dorsally in the STN (without covering any of the central/ventral STN), this may not have the same cognitive benefits (in terms of stopping control) as when a larger part of the STN is covered by the VTA, including a dorsal and more central/ventral area. Note that our experimental ventral stimulation did not extend to the most ventral limbic region of the STN, which would likely induce emotional side effects (Mallet et al. 2007; Okun et al. 2009; Accolla and Pollo 2019). Individual differences in the optimal balance between cognitive and motor benefits with clinical stimulation settings would need further study.

Beyond the beneficial findings of DBS on stopping control, clinical stimulation parameters, either at clinical electrode points or at (unilateral) contact points in ventral STN (Hershey et al. 2010), have shown a negative impact on the overall motor system’s threshold to act (proactive control), reflected by increases in impulsive errors and faster responses under conditions of motor and decision conflict (Jahanshahi et al. 2000; Hershey et al. 2004; Witt et al. 2004; Frank 2006; Campbell et al. 2008; Ballanger et al. 2009; Hershey et al. 2010; Wylie et al. 2010). Similarly, Georgiev and colleagues (Georgiev et al. 2016) showed that DBS STN especially induced impulsive errors on NoGo trials when the probability of the Go trials increased (Go/NoGo task), whereas there was no stimulation effect with lower Go probability, confirming a role for STN DBS in adjusting a proactive response threshold.

With respect to putative changes in response thresholds in our study, neither focal dorsal nor ventral STN stimulation differentially impacted the performance on Go signals compared with the OFF stimulation condition. Notably, other clinical DBS studies using the conventional stop task or a Go/NoGo task have not reported faster or more erroneous Go performance ON versus OFF DBS either (van den Wildenberg et al. 2006; Swann et al. 2011; Mirabella et al. 2012). However, to measure DBS-induced changes in proactive control on the Stop task (comparable to findings with DBS on motor or decision conflict), it would require a comparison of Go trial performance in a Stop versus a simple choice task, or manipulation of probabilities of Go and Stop trials. This allows measuring the DBS effect on regulating the response threshold in a context that requires heightened cognitive control versus a context with fewer demands on control. Interestingly, Mirabella et al. (2013) showed that these context-dependent proactive adjustments in stopping control are restored by the clinical DBS. To gain more insight into whether stimulation in the STN subregions could explain the mixed DBS effects on proactive inhibitory control, future studies with stimulation in STN subregions and the above-described context manipulations are recommended.

### STN Involvement in Global Versus Selective Inhibition

The STN connects two functionally important pathways of the basal ganglia that are involved in the inhibition of movement, that is, the indirect and hyperdirect pathways. The STN receives input from the external globus pallidus (GPe, indirect pathway) and direct input from several cortical areas (hyperdirect pathway) (DeLong 1990; Mink 1996; Nambu et al. 2002; Nambu 2004; Haynes and Haber 2013; Alkemade et al. 2015; Plantinga et al. 2018). The latter has increasingly received attention because it could be involved in implementing a short-latency signal from the cortex to recruit the STN (Nambu et al. 2002; Chen et al. 2020) to rapidly interrupt ongoing action processing, such as selectively suppressing a conflicting response tendency from interfering with action selection (“selective inhibition”) or abruptly stopping all actions in response to sudden changes in goals (“global inhibition”). Our previous work with a conflict task showed that focusing DBS in the dorsal STN, but not the ventral STN subregion, improved the selective inhibition of conflicting action impulses (van Wouwe et al. 2017). The current study (global inhibition) and our previous work with focused STN subregion stimulation support the notion that the relatively dorsal STN (and its associated cortical circuitry) is linked to the “selective inhibition of a specific action during” conflict control, whereas a more ventral associative STN subregion is linked to the “global inhibition signaling” involved in stopping control (Aron et al. 2016). In line with the conceptual and behavioral dissociation of these forms of control (Friedman and Miyake 2004), the current work provides novel evidence that we can modulate dissociable forms of control when stimulation is applied to the focused subregions of the STN, a small but significant structure in the broader frontal–striatal network.

In addition to being linked to the IFC/preSMA and the connected STN circuitry, global inhibition has also been suggested to rely upon a right-lateralized network and right STN (for a review, see Aron et al. 2014), although studies with clinical DBS have shown that only bilateral DBS STN restores stopping control (Mirabella et al. 2013; Mancini et al. 2018) and there is no difference in the right- and left-onset PD patients with respect to the inhibitory control impairments (Mirabella et al. 2017). The current results do not seem to suggest either that there is a lateralization effect, that is, we did not find a differential effect of DBS across response hands with respect to the stopping latencies, although ultimately the lateralization effect would need to be tested with unilateral stimulation across the STN subregions.

Beyond the current study, few studies have directly measured or stimulated in the ventral STN subregion during the stop-signal task performance. An exception is a study that directed clinical DBS at the most ventral contact point of the electrode lead and reported no modulation of stopping performance (Greenhouse et al. 2011). Notably, several of the ventral contact points fell outside of the STN or appeared situated in the most anterior ventral tip of the STN, which has been linked to limbic circuitries. Thus, it is difficult to compare their results with the current study. Perhaps more relevant is a recent primate study (Pasquereau and Turner 2017) that demonstrated increased STN firing in a ventromedial subregion during successful stopping. Similarly, Chen et al. (2020) found that when the cortical IFG activity strongly correlated with the STN activity, this was associated with more successful stopping in PD patients intraoperatively. Also, intraoperative ventral STN stimulation evoked larger potentials in IFG compared with dorsal stimulation. Our work converges on the significance of a ventral STN subregion in human stopping control.

We applied a novel stimulation strategy to restrict the projected area of STN tissue activation (van Wouwe et al. 2017). Although our approach provides better fidelity in stimulating a specific STN subregion compared with clinical DBS settings, the experimental stimulation could still produce overlapping fields of activation. It is also important to recognize that the “exact” demarcation of functional subterritories across the STN, as found in the current study, remains uncertain and could be a gradient similar to the structural gradient of cortical innervations from motor, associative, and limbic areas traversing, respectively, the dorsolateral to ventromedial STN (Keuken et al. 2012; Plantinga et al. 2018). Technological developments in the electrode lead registration (Horn et al. 2017; Horn et al. 2019) combined with white matter tractography and advanced models to estimate the volume of tissue activated will enable future studies to pinpoint the functional and structural gradients of the frontal–basal ganglia circuits involved in stopping and conflict control.

We acknowledge that the underlying mechanism of DBS is still under debate and likely has several electrical and neurochemical effects on both local and network-wide levels; for example, stimulation could act similar to a functional lesion by inhibiting neurons near the electrode, elicit antidromic action potentials to cortex, or reduce overactive beta-band oscillations between the cortex and STN (Chiken and Nambu 2016; Herrington et al. 2016). These explanations are likely overlapping, and their relevance for inhibitory control remains to be investigated in future neurophysiological studies.

In the current study, DBS effects on inhibitory control could be explained by modulations of both hyperdirect and indirect pathways. The hyperdirect cortical projections to STN could be involved in providing a fast, global inhibitory signal to pause or halt all ongoing action plans to allow more time to respond, and stimulation of the STN could modulate input from these cortical projections.

The STN stimulation effect on inhibitory control could be additionally attributed to disrupted information flow in the indirect pathway (through striatum and GPe) (Reese et al. 2011). A recent model (Sajad et al. 2019) showed that cortical modulations of the indirect pathway (through gain- and error-based signals from medial prefrontal cortex to striatum) regulate stopping performance without the involvement of a hyperdirect pathway. Furthermore, the indirect pathway could be involved in implementing a more selective, gradually build up inhibitory control signal to suppress a conflicting response impulse.

However, the relative contribution and temporal involvement of hyperdirect and indirect pathways to inhibitory control need further investigation. Moreover, given that clinical stimulation likely incorporates some part of each of these subregions, it remains uncertain which aspects of DBS stimulation and their effect on inhibitory control are most closely tied to the beneficial clinical effects.

## Conclusion

In conclusion, we provide pivotal evidence that stimulation in the ventral STN subregion improves global action stopping relative to stimulation in the dorsal STN. An improved understanding of which functional circuits are stimulated and how this affects inhibitory control may provide the groundwork to more precise stimulation strategies and for closed-loop stimulation to optimize motor and cognitive deficits in PD and related basal ganglia disorders.

## Notes

Anonymized data will be available on reasonable request by a qualified investigator. *Conflict of Interest*: Dr. Dawant is a founder and equity holder in Neurotargeting, LLC., that licenses some of the technology from Vanderbilt University described in this article. Dr. Phibbs has done consulting for Boston Scientific, Medtronic and Teva. There are no other conflicts of interest to report.

## Funding

This work was supported by the National Institutes of Health R01NS100996 (to J.S.N. and N.C.v.W.), RO1NS095291 (to B.M.D.) and by the Vanderbilt University Medical School Program UL1 RR 024975 (to A.M.L.).
